# The Impact of Dietary Knowledge on Health: Evidence from the China Health and Nutrition Survey

**DOI:** 10.3390/ijerph18073736

**Published:** 2021-04-02

**Authors:** Yangyang Sun, Daxin Dong, Yulian Ding

**Affiliations:** School of Business Administration, Faculty of Business Administration, Southwestern University of Finance and Economics, Chengdu 611130, China; sunyangyang@smail.swufe.edu.cn (Y.S.); dongdaxin@swufe.edu.cn (D.D.)

**Keywords:** dietary knowledge, self-rated health, heterogeneity, China Health and Nutrition Survey

## Abstract

Promoting a healthy diet through education is part of the Healthy China 2030 action plan. However, studies examining how dietary knowledge affects public health in China are sparse. This study employs multiple waves of the China Health and Nutrition Survey (CHNS) data to examine the impacts of dietary knowledge on Chinese adults’ health, with a particular emphasis on how the impacts of dietary knowledge vary across different demographic groups. Moreover, we contribute to the literature by incorporating the spouse’s dietary knowledge into the analysis framework to inspect the relationship between a spouse’s dietary knowledge and an individual’s health. Our results indicate that dietary knowledge significantly improves an individual’s health status. However, there is no evidence that an individual’s health is influenced by his/her spouse’s dietary knowledge. Moreover, we find that individuals with a lower level of education and rural residents benefit more from increasing dietary knowledge. Policy implications of this study are also discussed.

## 1. Introduction

With rapid economic growth, many Chinese have experienced significant changes in their lifestyle. The prevalence of overweight among Chinese adults aged from 18 to 75 years has increased from 14.6% in 1992 to 45.4% in 2011 [1]. In the meantime, the obesity rate has almost tripled from 5.2% to 15.1% [1]. Obesity raises the risk of many chronic diseases, such as hypertension, cardiovascular diseases, and diabetes. China spends approximately CNY 24.35 billion (USD 3.24 billion) each year to treat obesity-related diseases, accounting for 2.46% of China’s annual healthcare expenditures [2]. Increased consumption of energy-dense foods with high levels of sugar and saturated fats, and reduced physical activity, are often considered the major drivers of weight gain [2]. Consequently, there is growing interest in promoting healthy eating to reduce obesity and diet-related diseases.

As a national plan to promote public health, the Healthy China 2030 blueprint was released in 2016, and a corresponding action plan was later announced by the Chinese State Council in 2019. The action plan outlined specific goals to be achieved between 2020 and 2030, including promoting healthy diets through education [3]. 

Previous studies show that a lack of dietary knowledge can lead to poor eating habits and bad health conditions [4]. Although improving the dietary knowledge of the general public helps reduce obesity and diet-related chronic diseases [5,6], the impacts of dietary knowledge on health vary across people with different social and economic characteristics [7,8,9,10]. Studies examining how dietary knowledge affects public health in China are sparse. Yang et al. [11] find dietary knowledge leads to better self-rated health of Chinese adults based on the 2015 China Health and Nutrition Survey (CHNS) data. Since China is a vast country with a huge population, it is of importance to investigate the heterogeneous impacts of dietary knowledge on health across different groups of people. Moreover, there is growing interest examining peer effects in the context of healthy eating [12]. As family members spend much time together and usually have the same or similar dietary knowledge and lifestyle habits, such as smoking [13,14] and alcohol consumption [15,16], how a family member’s dietary knowledge might affect an individual’s health is worth investigating. Therefore, this study employs multiple waves of the CHNS data to examine the impacts of dietary knowledge on Chinese adults’ health, with a particular emphasis on how the impacts of dietary knowledge on health vary across different demographic groups. Moreover, we incorporate the spouse’s dietary knowledge into the analysis framework to inspect the relationship between a spouse’s dietary knowledge and an individual’s health, which is considered less in previous studies. Our findings may provide insights on designing more effective policies to promote public health in China.

The remainder of this paper is organized as follows. Section 2 introduces related research. Research design, including data processing, variable selection, and model construction, is described in Section 3. Section 4 reports the estimation results and robustness checks. Conclusions and policy implications are presented in Section 5 and Section 6, respectively.

## 2. Literature Review

Previous studies show that poor dietary habits (e.g., being addicted to energy-dense nutrient-poor foods) significantly increase chronic diseases and health problems [17,18]. These habits are partly due to low levels of nutrition knowledge. Wang et al. [19] argue that individuals’ dietary knowledge may change their food preferences and eating habits, and consequently reduce obesity prevalence and other health problems [20,21,22]. Kunitomo et al. [23] show that higher levels of nutrition knowledge significantly decrease the consumption of sugar-sweetened beverages, while Spillmann and Siegrist [24] report that improved nutrition knowledge increase fruit and vegetable intake. De Vriendt et al. [8], Petrovici and Ritson [25] find that an individual’s level of nutrition knowledge mainly depends on his/her education, age, and occupation. Some scholars argue that more knowledge of diet usually leads to better health [11,26]. However, there are also studies suggesting that dietary knowledge is not translated into healthier food choices [27], and so has no positive impacts on health [28,29].

A growing body of the literature recognizes that the effect of dietary knowledge on health is affected by individual characteristics. For example, it has been noted that the impact of dietary knowledge on health may vary between different income groups. High-income groups are more likely to have access to information on healthy eating [30], and to transform dietary knowledge into actions [31]. It is relatively difficult for low-income groups to change their dietary patterns due to budget constraints and healthy food accessibility, even though they understand dietary knowledge. Clement and Bonnefond [5] show that people with high levels of education and living in cities tend to have higher levels of dietary knowledge and healthier food preferences. Mader et al. [32] find that there is no relationship between income and dietary quality, but individuals with high education levels are less corpulent than others. Furthermore, some studies have demonstrated that peers and friends play an important role in influencing an individual’s food preferences and eating behaviors [33,34]. Family members, spouses, in particular, spend a long time with each other, and their lifestyles and health behaviors influence each other. Clark and Etile [35] show that obesity of spouses tends to be contagious. The weight of one spouse significantly affects the weight of the other. In addition, there are spillover effects in health behaviors between spouses. If one quits smoking, the chance that the other spouse smokes decreases by up to 30% [36].

Scholars have long debated the impacts of dietary knowledge on health conditions [19,20,37]. As the impacts vary across people with different characteristics, it is important to separate different demographic groups to identify how the impacts differ among these groups. Given that studies examining the impacts of dietary knowledge on public health in China are limited, we employ the CHNS data to investigate the heterogeneity in the impacts of dietary knowledge on health among the Chinese adults. We also investigate how an individual’s health might be affected by his/her spouse’s dietary knowledge. Contributing to the literature, we divide spouses into female spouses (i.e., wives) and male spouses (i.e., husbands), and examine whether the impact of a wife’s dietary knowledge on the health of her husband differs from the impact of a husband’s dietary knowledge on the health of his wife.

## 3. Data, Variables and Model

### 3.1. Data

**Figure A1:**
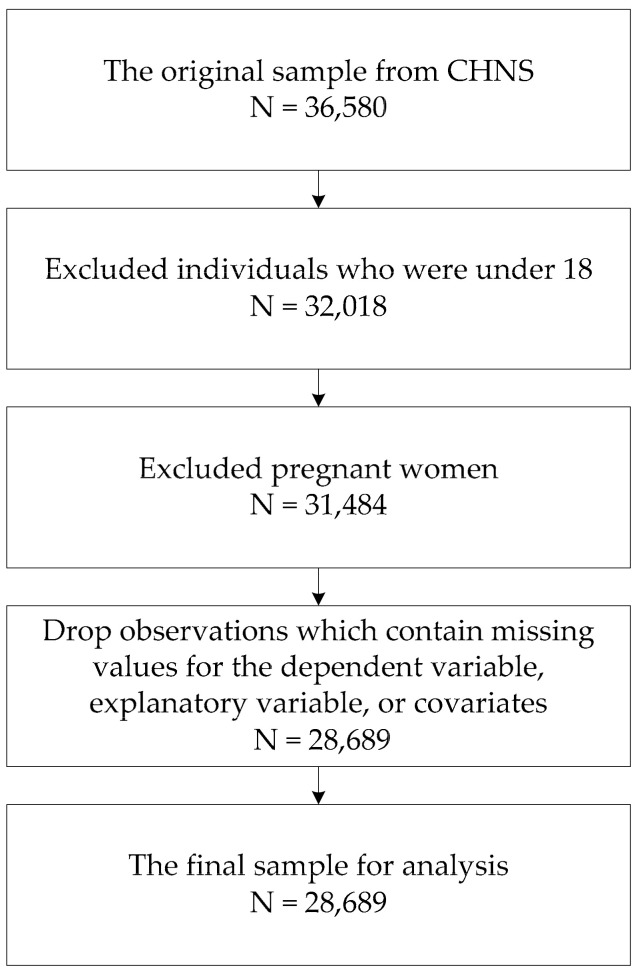
The sample selection process.

We use the China Health and Nutrition Survey (CHNS) data. CHNS is an international collaborative project designed to monitor how China’s social and economic transformation affects the health of the Chinese population. The sample was drawn from 15 provinces and municipal cities using a multistage, random cluster process. The CHNS was conducted ten times during the time period from 1989 to 2015. The survey team started collecting information on dietary knowledge in 2004, and the data of self-rated health are available only in 2004, 2006, and 2015. Consequently, we employ three waves of CHNS data collected in 2004, 2006, and 2015, respectively. Although the structure of our data is independently pooled cross-section, some individuals were repeatedly selected, accounting for about 16 percent of the whole sample. Considering that children and adolescents may not understand dietary knowledge due to limited cognitive abilities, we excluded individuals who were under 18. We also excluded pregnant women as they generally pay more attention to nutrition intake. The observations with a missing value in any of the variables were also deleted. The final sample consists of 28,689 observations (9364 in 2004, 9289 in 2006, and 10,036 in 2015). The sample selection process was listed in Figure A1 in the Appendix A.

### 3.2. Variables

#### 3.2.1. Dependent Variable

We use an individual’s self-reported health status (*health_self*) as the indicator of his/her health condition. Although self-rated health is simple and subjective, it is a comprehensive evaluation of an individual’s health and has been proven to successfully predict mortality and disability [38]. Many previous studies have also used self-rated health status to measure the health of individuals [39,40,41]. In the CHNS, the following question was asked: “Right now, how would you describe your health compared to that of other people your age?” The respondents chose one of five options: “1. Very good”, “2. Good”, “3. Fair”, “4. Bad”, “9. Unknown”. In the 2015 survey, a new option was added, “5. Very bad”. As the proportion of respondents who chose “1. Very good” and “5. Very bad” were very small, we grouped “Very bad” and “Bad” together and referred to these groups as “Bad”. We also grouped “Very good” and “Good” together and referred to these groups as “Good”. Respondents who chose “9. Unknown” were excluded from the analyses. We reversed the option sequence “2, 3, 4” to “3, 2, 1”, so a larger number indicates better health.

#### 3.2.2. Core Explanatory Variable of Interest

The core explanatory variable is the respondents’ dietary knowledge. We measure the respondents’ dietary knowledge based on their attitudes toward 12 diet-related statements, and this approach has been widely used to measure the levels of dietary knowledge in the literature [31,42,43]. The 12 diet-related statements are listed in Table A1 in the Appendix A. Respondents’ attitudes toward each statement are measured on a 5-point Likert scale (1 = strongly disagree, 5 = strongly agree), with an additional option of “9. unknown”. Based on the WHO [44] criteria, we divided the statements into “True” and “False”. For the “True” statements, 1 point was given if a respondent chose “strongly disagree” or “disagree”, and 3 points were given if a respondent chose “agree” or “strongly agree”. For the “False” statements, 3 points were given if a respondent chose “strongly disagree” or “disagree”, and 1 point was given if a respondent chose “agree” or “strongly agree”. Respondents received 2 points if they chose “neutral” or “unknown” regardless of the “True” or “False” statements. Respondents’ dietary knowledge scores were calculated by adding up their scores for all 12 items. A higher score indicates a higher level of dietary knowledge. 

#### 3.2.3. Covariates

Previous studies show that the impacts of dietary knowledge on health vary across people with different personal characteristics. We controlled these characteristics in our analyses. The definitions and summary statistics of the variables are presented in Table 1. The average self-rated health level is 2.506, which means that on average respondents self-rated their health status as “Fair”. The score of respondents’ dietary knowledge varies from 17 points to 36 points, with a mean score of 31.29 points, suggesting that respondents’ dietary knowledge levels vary greatly. 

### 3.3. Model

In this paper, self-rated health status is used to measure individual health. The ordered logit model has been widely used to analyze discrete choice data [39,40,41]. However, using the ordered logit model requires that the Parallel Regression Assumption (PRA) be met. We use the Brant-Test to test this assumption (test on equality of all regression coefficients from all binary logistic regressions). The test result shows that the PRA is violated (χ^2^ = 585.3, the statistical significance is at 0.01%). Consequently, we use a generalized ordinal logit model to examine the influence of dietary knowledge on health status. Compared with the traditional ordered logit model, the generalized ordinal logit model relaxes the assumption of parallel lines. Moreover, the generalized ordinal logit model can analyze how the influence of the independent variable on the dependent variable may change as the threshold of the latent variable changes. The model takes the following form:

(1) $$P(Y_{i}>j)=g\left(X\beta_{j}\right)=\frac{exp(\alpha_{j}+X_{i}\beta_{j})}{1+\left[exp(\alpha_{j}+X_{i}\beta_{j}\right)]},j=1,2,\ldots ,M-1$$

where *M* is the number of categories of the ordinal dependent variable. The probabilities that *Y* will take on each of the values 1, ..., *M* are equal to

(2) $$P\left(Y_{i}=1\right)=1-g\left(X_{i}\beta_{1}\right)\;P\left(Y_{i}=j\right)=g\left(X_{i}\beta_{j-1}\right)-g\left(X_{i}\beta_{j}\right),j=2,\ldots ,M-1\;P\left(Y_{i}=M\right)=g\left(X_{i}\beta_{Μ-1}\right)$$

In Equation (1), *Y_i_* is the status that respondent *i* rated his/her health as *M* (Bad (*M* = 1); Fair (*M* = 2); Good (*M* = 3)). *j* is the *j*-th category level of the ordered multi-categorical variable. If *j* = 1, category 1 is contrasted with categories 2, and 3; if *j* = 2, the contrast is between the first two categories (1 and 2) and the third category (3). If the estimated coefficients corresponding to each category are equal, the generalized ordinal logit model can be transformed into the traditional ordered logit model. *X_i_* contains the independent variable (*diet_knowledge*) and covariates (including *gender*, *ethnic*, *age*, *married*, *smoking*, *alcohol*, *education*, *lnper_income,* and *city)*. We also control the province-fixed effects and time-fixed effects. *α* and *β* are the parameters to be estimated. The estimation results are reported in Table 2.

## 4. Results

In this section, we report the estimation results of our regression model. In Section 4.1, we examine how dietary knowledge affects the probability of having a good health status. In Section 4.2, we investigate whether an individual’s health is influenced by his/her spouse’s dietary knowledge. In Section 4.3, we analyze the heterogeneity among different socio-demographic groups. Finally, we report robustness checks in Section 4.4.

### 4.1. The Impact of Dietary Knowledge on Health Status

We z-standardized “*dietary_knowledge*” before estimation, so that we will be able to know, if dietary knowledge changes by one standard deviation, how an individual’s health status may change. Table 2 shows the estimation results of the generalized ordinal logit model (Equations (1) and (2)). The coefficients in column (i) are estimated by comparing individuals with a self-evaluated health status as “Bad” with those who rated their health status as “Fair” or “Good”. The coefficients in column (ii) are estimated by comparing individuals with a self-evaluated health status as “Bad” or “Fair” with those who rated their health status as “Good”. As shown in columns (i) and (ii), the estimated coefficients on *diet_knowledge* are 0.114 and 0.102, which are both statistically significant at the 0.1% level. This shows that dietary knowledge has a positive effect on self-rated health. As the parameters of the generalized ordinal logit model are not directly comparable, we calculated the average marginal effects of dietary knowledge on self-rated health, and reported the marginal effects in columns (iii)—(v). As can be seen in these columns, if dietary knowledge increases by one standard deviation, on average, the probability that the self-rated health status is “Bad” and “Fair” will decrease by 0.7 percentage points and 1.5 percentage points, respectively; in contrast, the probability that the self-rated health status is “Good” will increase by 2.2 percentage points.

The coefficients on most of control variables are also statistically significant. As can be seen in Table 2, males are more likely to rate their health status as “Good” than females. The Han Chinese tend to have better self-rated health status than other ethnic groups. The older people are more likely to rate their health status as “Bad” or “Fair”, which is consistent with our expectation as an individual’s health condition tends to worsens when he/she ages. Our results also show that an individual’s health improves as his/her education or income level improves, and that on average, the self-evaluated health status of rural residents is better than that of urban residents. We find no evidence that one’s marital status affects his/her self-rated health. The finding that *smoking* and *alcohol* both have significant positive effects on self-rated health status is unexpected. Previous medical studies demonstrated that smoking and alcohol consumption adversely affect health and induce chronic diseases [45,46,47]. However, several studies identified that the self-rated health conditions of smokers and drinkers tend to be biased upwards [11,48]. There are two possible explanations: one is that those who are addicted to smoking and alcohol consumption generally believe that their health is better and that smoking and alcohol consumption have a less negative impact on their health [49]; the other is that smoking and alcohol consumption may relieve psychological pressure and make people happy [50], leading to a better self-evaluation of health status.

### 4.2. The Impact of Spouse’s Dietary Knowledge on Individual Health Status

In this subsection, we examine whether the dietary knowledge of an individual’s spouse affects his/her health status. We only retain the samples whose marital status is “married” for analyses in this section, including 22,144 observations. The variable *diet_knowledge_sp* is included in the regression model.

Estimated coefficients are reported in Table 3. The results in columns (i) and (ii) show that individuals’ dietary knowledge has a significant positive impact on her/his health status. The estimated coefficient of *diet_knowledge* is statistically significant. The estimated marginal effects are reported in columns (iii)—(v). In particular, if dietary knowledge increases by one standard deviation, on average, the probability that the self-rated health status is “Bad” decreases by 0.4 percentage points while the probability of rating one’s health as “Good” increases by 1.7 percentage points.

The dietary knowledge level of the spouse, however, has no significant impact on an individual’s health conditions, as reported in Table 3. Several prior studies reported that the health behaviors of spouses affect each other, and there are spillover effects [36,51]. We employed the data from Chinese households and measured individual health level with self-rated health, and found that an individual’s health condition does not benefit from his/her spouse’s dietary knowledge. This finding can be explained from two aspects. First, although couples often make decisions together, they may decide what and how much to eat independently. Moreover, during working days, individuals often eat in their workplaces rather than at home. Second, the communication efficiency between spouses may be low [52]. Traditionally, males take more financial responsibilities and females are mainly responsible for housework and childcare, so males are usually in a strong position in a family [53]. Now, females increasingly participate in the labor market, and they also share the financial burden of the family. Their authority in the family has also increased [54]. The conflicts between tradition and changing gender roles may decrease the efficiency of communication between spouses [55]. Therefore, we identify no significant association between an individual’s health and his/her spouse’s dietary knowledge. 

In China, wives are mainly responsible for preparing food in the home. We conjecture that the effect of wife’s dietary knowledge on husband’s health may be different from that of husband’s dietary knowledge on wife’s health. To analyze this, we divided the spouse into female spouses (i.e., wives) and male spouses (i.e., husbands). The estimated results are listed in Table 4 and Table 5. We found that an individual’s health was only correlated with his/her own dietary knowledge and there was no relationship between an individual’s health and the dietary knowledge of his/her spouse (i.e., wife or husband). This finding suggests that to improve one’s health, the most important thing is to increase one’s own dietary knowledge.

### 4.3. Heterogeneity Analysis

Dietary knowledge differs across people. Xu et al. [56] conclude that there are significant differences in dietary knowledge among individuals with different incomes and from different regions. Xie and Mo [57] find that individuals with a higher level of education tend to pay more attention to learning dietary knowledge and preventing health risks. Similarly, Zhao et al. [58] demonstrate that individuals in different socio-demographic groups reacted differently after receiving their health information. In this subsection, we group individuals based on their education, income, and residential area, and analyze the heterogeneous effects of dietary knowledge on health among these different groups.

We compare between high- and low-education groups, high- and low-income groups, as well as rural and urban residents. Individuals with an education level above the sample median (i.e., lower middle school degree) are classified into the high-education group. Similarly, the high-income group consists of individuals with an annual income above the sample median (approximately CNY 8745 (USD 1162)). We also divide the whole sample into the rural (*city* = 0) group and urban (*city* = 1) group.

Table 6 reports the results of heterogeneity analyses. As shown in columns (i) and (ii) of Table 6, dietary knowledge has a significant positive impact on self-rated health for the low-education group, but the impact is not statistically significant (except for self-rated health as “Good”) for the high-education group. This suggests that improving dietary knowledge is more important for improving the health of the low-education group. A possible explanation is that the low-education group generally has a lower level of dietary knowledge, so there is more room for individuals in this group to improve their eating habits as they gain new dietary knowledge. However, individuals in the high-education group already understand the importance of healthy eating; their health problems are probably more caused by lifestyle or work style, such as frequent meals in fast-food restaurants. Therefore, increasing dietary knowledge has no impact on their health.

The results in columns (iii) and (iv) of Table 6 reveal that the impacts of dietary knowledge on health are similar between the low- and high-income groups. As can be seen in column (v) of Table 6, dietary knowledge has a significantly positive effect on the self-rated health of rural residents. If dietary knowledge increases by one standard deviation, the probability that rural residents rate their health as “Good” will increase by 3.2 percentage points. Results in column (vi), however, show that dietary knowledge has no significant impact on the self-rated health of urban residents. Urban residents may have relatively more dietary knowledge, but the food environment (availability of all sorts of processed foods and restaurants) makes it more difficult for them to follow dietary knowledge and eat healthily. Thus, in urban areas, the role of dietary knowledge in promoting health is not significant. 

Based on the results in Table 6, we can conclude that dietary knowledge has a greater effect on improving the health of individuals with low education and living in the rural area.

### 4.4. Robustness Check

We conducted robustness checks by changing the measurement of dietary knowledge, the sample, and the model specification, and re-estimated the model.

#### 4.4.1. Changing the Measurement of Dietary Knowledge

Previously, we measured the level of dietary knowledge by calculating the scores of answering the relevant questions in the questionnaire. Now, we use the proportion of correct answers to measure dietary knowledge based on the CHNS questionnaire (see Table A1 in the Appendix A for details). The calculation method is as follows: For “True” statements, the answer “agree” or “strongly agree” is recorded as correct, otherwise the answer is wrong; for “False” statements, the answer “strongly disagree” or “disagree” is recorded as correct, and the answer is wrong otherwise. We then use the percentage of correct answers as a proxy variable for the level of dietary knowledge. A higher percentage of correct answers indicates a higher level of dietary knowledge. Before regression estimation, the variable of this new measurement of dietary knowledge was z-standardized. The results of Part A in Table A2 in the Appendix A show that dietary knowledge has a significantly positive impact on self-evaluated health status. If the proportion of correct answers increases by one standard deviation, on average, the probability of respondents’ self-evaluated health as “Good” will increase by 2.8 percentage points. The results from the model including the spouse’s dietary knowledge are shown in Part B of Table A2 in the Appendix A. As before, we find that one’s health is affected by his/her dietary knowledge, and that spouse’s dietary knowledge has no influence on an individual’s health.

#### 4.4.2. Changing the Sample

Individuals who suffer from hypertension or diabetes may pay more attention to dietary knowledge as well as lifestyles. Including these individuals in our study sample may lead to biased estimates. Therefore, now we exclude individuals with high blood pressure or diabetes. In the CHNS, the respondents were asked: “Has a doctor ever told you that you suffer from high blood pressure?”, “Has a doctor ever told you that you suffer from diabetes?” We dropped those who answered “Yes” and “Unknown” from the sample. The results of Part A in Table A3 in the Appendix A show that after excluding samples with high blood pressure or diabetes, dietary knowledge still has a significant positive impact on individual health. The probability of self-rated health as “Good” increases by 2.4 percentage points if dietary knowledge increases by one standard deviation. Including the dietary knowledge of one’s spouse in the model, the coefficient on dietary knowledge is still significantly positive as shown in Part B of Table A3 in the Appendix A. Interestingly, we identified a significant positive impact of spouse’s dietary knowledge on one’s health in this sample, suggesting that more research is needed to verify whether an individual’s health is influenced by his/her spouse’s dietary knowledge.

#### 4.4.3. Changing the Model Specification

Considering that smoking and alcohol consumption might interact with an individual’s dietary knowledge and self-rated health, we removed the covariates *smoking* and *alcohol*, and re-estimated the model. The results of the reduced model are reported in Table A4 in the Appendix A. As can be seen in Table A4, individuals with a higher level of dietary knowledge are more likely to rate their health as “Good”, which is consistent with the results in Table 2. Overall, the robustness checks in Table A2, Table A3 and Table A4 in the Appendix A support the finding that improving dietary knowledge contributes to better health.

## 5. Conclusions

There is growing interest in promoting healthy eating to reduce food-related chronic diseases worldwide. We employ the generalized ordinal logit model to analyze how an individual’s self-rated health status is influenced by his /her dietary knowledge as well as the dietary knowledge of the spouse, using multiple waves of the CHNS data. The results show that dietary knowledge has a significantly positive impact on an individual’s self-rated health. If dietary knowledge increases by one standard deviation, the probability of self-evaluated health as “Bad” or “Fair” decreases by 0.7 percentage points and 1.5 percentage points, respectively, while the likelihood of self-rated health as “Good” increases by 2.2 percentage points. Considering that spouses usually have similar lifestyles [13], this study also examines the effect of a spouse’s dietary knowledge on an individual’s health. We find no evidence that an individual’s health is affected by his/her spouse’s dietary knowledge. We also explored the effects of female spouses and male spouses separately. Our results show that a wife’s dietary knowledge does not affect the health of her husband and vice versa. A possible explanation is that, although couples make many decisions together, they may often decide what and how much to eat independently.

Findings on the impact of dietary knowledge on health differ among different studies, which may be due to ignoring the heterogeneity of the samples [23,28,29]. Our heterogeneity analyses suggest that the impacts of dietary knowledge on health are stronger for individuals with a lower level of education and for rural residents. When dietary knowledge increases by one standard deviation, the probability of self-rated health as “Good” will increase by 2.9 percentage points for the low-education group and 1.1 percentage points for the high-education group. Compared with the high-education groups, the low-education group benefit more from acquiring dietary knowledge. We find that the impact of dietary knowledge on health is not significantly different between the low-income and high-income group, which is consistent with the latest study [32]. Our results also show that dietary knowledge has a bigger effect on the health of rural residents than urban residents, which may be due to their different lifestyles.

## 6. Policy Implications

The policy implications of our findings are as follows: first, dietary knowledge contributes to better health, so it is important to increase the dietary knowledge of the general public in China. The government could take measures to educate the general public about nutrition knowledge and healthy eating. Popularizing the dietary guidelines for the Chinese residents may also help increase individuals’ dietary knowledge, leading to improved public health. Second, our results reveal that the impacts of dietary knowledge on health vary across people. Individuals with a lower level of education and rural residents benefit more from improved dietary knowledge. Therefore, the government should focus more on educating these groups as dietary knowledge plays a greater role in improving the health of individuals in these groups. Moreover, dietary knowledge has no impact on the health of the high-education group and urban residents, suggesting that actions other than improving dietary knowledge are needed to promote healthy eating among these groups, for example, creating a healthy food environment.

This study has several limitations. Our study examined the impacts of dietary knowledge on health, but did not analyze the underlying mechanism through which the influences occur, i.e., we did not know how an increase in dietary knowledge may lead to better health. Unfortunately, in this study, we cannot conduct the mechanism analyses due to data limitations. Future studies may shed light on how increased dietary knowledge improves health as well as why the impacts of dietary knowledge on health are heterogeneous.

## Figures and Tables

**Table 1:** Summary statistics of the analytical samples.

Variables	Definition	N	Mean	SD	Min	Max
*health_self*	1—Bad; 2—Fair; 3—Good	28,689	2.506	0.626	1	3
*diet_knowledge*	Summation of the scores for 12 statements in the questionnaire	28,689	31.29	3.252	17	36
*gender*	1 = Male; 0 = Female	28,689	0.489	0.500	0	1
*ethnic*	1 = Han; 0 = Other ethnic	28,689	0.886	0.318	0	1
*age*	Age of the person	28,689	48.95	15.12	18	97
*married*	1 = Yes; 0 = No	28,689	0.850	0.357	0	1
*smoking*	1 = Yes; 0 = No	28,689	0.272	0.445	0	1
*alcohol*	1 = Yes; 0 = No	28,689	0.317	0.465	0	1
*education*						
*Illiteracy*	1 = Yes; 0 = No	28,689	0.149	0.356	0	1
*Lower middle school degree and below*	1 = Yes; 0 = No	28,689	0.532	0.499	0	1
*Upper middle school degree or vocational degree*	1 = Yes; 0 = No	28,689	0.228	0.420	0	1
*University degree or higher*	1 = Yes; 0 = No	28,689	0.091	0.287	0	1
*lnper_income*	Per capita annual household income inflated to 2015, CNY, in logarithm	28,689	8.982	1.197	0.594	13.94
*city*	1 = Urban; 0 = Rural	28,689	0.372	0.483	0	1

**Table 2:** Estimation results of the effect of dietary knowledge on health based on the generalized ordinal logit model.

Variables	Baseline Model		Marginal Effect		
			*health_self* = 1 Bad	*health_self* = 2 Fair	*health_self* = 3 Good
	(i)	(ii)	(iii)	(iv)	(v)
diet_knowledge	0.114 ***	0.102 ***	−0.007 ***	−0.015 ***	0.022 ***
	*(0.0233)*	*(0.0131)*	*(0.0014)*	*(0.0028)*	*(0.0029)*
gender	−0.108	0.095 **	0.007	−0.028 ***	0.021 **
	*(0.0594)*	*(0.0334)*	*(0.0037)*	*(0.0072)*	*(0.0073)*
ethnic	−0.066	0.173 ***	0.004	−0.042 ***	0.038 ***
	*(0.0873)*	*(0.0460)*	*(0.0054)*	*(0.0102)*	*(0.0101)*
age	−0.038 ***	−0.033 ***	0.002 ***	0.005 ***	−0.007 ***
	*(0.0018)*	*(0.0010)*	*(0.0001)*	*(0.0002)*	*(0.0002)*
married	0.086	0.006	−0.005	0.004	0.001
	*(0.0639)*	*(0.0374)*	*(0.0039)*	*(0.0080)*	*(0.0082)*
smoking	0.115	0.069 *	−0.007	−0.008	0.015 *
	*(0.0676)*	*(0.0353)*	*(0.0042)*	*(0.0077)*	*(0.0078)*
alcohol	0.510 ***	0.164 ***	−0.032 ***	−0.004	0.036 ***
	*(0.0670)*	*(0.0333)*	*(0.0042)*	*(0.0073)*	*(0.0073)*
education					
Lower middle school degree and below	0.374 ***	0.265 ***	−0.027 ***	−0.034 ***	0.060 ***
	*(0.0685)*	*(0.0420)*	*(0.0053)*	*(0.0095)*	*(0.0096)*
Upper middle school degree or vocational degree	0.626 ***	0.424 ***	−0.041 ***	−0.055 ***	0.095 ***
	*(0.0913)*	*(0.0493)*	*(0.0060)*	*(0.0110)*	*(0.0112)*
University degree or higher	0.766 ***	0.494 ***	−0.047 ***	−0.063 ***	0.110 ***
	*(0.1402)*	*(0.0655)*	*(0.0076)*	*(0.0144)*	*(0.0146)*
lnper_income	0.176 ***	0.123 ***	−0.011 ***	−0.016 ***	0.027 ***
	*(0.0218)*	*(0.0126)*	*(0.0014)*	*(0.0027)*	*(0.0028)*
city	−0.090	−0.140 ***	0.006	0.025 ***	−0.031 ***
	*(0.0524)*	*(0.0279)*	*(0.0032)*	*(0.0061)*	*(0.0061)*
Constant	3.028 ***	0.848 ***			
	*(0.2964)*	*(0.1499)*			
Province—Fixed Effect	Yes		Yes		
Year—Fixed Effect	Yes		Yes		
Pseudo R^2^	0.071				
Log pseudolikelihood	–23,259.046				
Observations	28,689		28,689		

Note: (1) ***, **, and * represent the significance levels of 0.1%, 1%, and 5%, respectively. (2) In columns (i) and (ii), the standard errors are reported in parentheses. In columns (iii)—(v), the delta-method standard errors are reported in parentheses.

**Table 3:** Estimation results of the effect of spouse’s dietary knowledge on health based on the generalized ordinal logit model.

Variables	Baseline Model		Marginal Effect		
			*health_self* = 1 Bad	*health_self* = 2 Fair	*health_self* = 3 Good
	(i)	(ii)	(iii)	(iv)	(v)
diet_knowledge	0.074 *	0.074 ***	−0.004 *	−0.012 **	0.017 ***
	*(0.0328)*	*(0.0177)*	*(0.0020)*	*(0.0039)*	*(0.0039)*
diet_knowledge_sp	−0.034	0.023	0.002	−0.007	0.005
	*(0.0328)*	*(0.0176)*	*(0.0020)*	*(0.0039)*	*(0.0039)*
gender	−0.057	0.106 **	0.003	−0.027 ***	0.024 **
	*(0.0691)*	*(0.0385)*	*(0.0042)*	*(0.0083)*	*(0.0085)*
ethnic	−0.064	0.154 **	0.004	−0.038 ***	0.034 **
	*(0.1025)*	*(0.0526)*	*(0.0062)*	*(0.0117)*	*(0.0117)*
age	−0.043 ***	−0.036 ***	0.003 ***	0.005 ***	−0.008 ***
	*(0.0023)*	*(0.0012)*	*(0.0001)*	*(0.0003)*	*(0.0003)*
smoking	0.128	0.070	−0.008	−0.008	0.015
	*(0.0769)*	*(0.0395)*	*(0.0046)*	*(0.0087)*	*(0.0088)*
alcohol	0.502 ***	0.177 ***	−0.030 ***	−0.009	0.039 ***
	*(0.0752)*	*(0.0374)*	*(0.0046)*	*(0.0083)*	*(0.0083)*
education					
Lower middle school degree and below	0.382 ***	0.283 ***	−0.027 ***	−0.038 ***	0.065 ***
	*(0.0788)*	*(0.0481)*	*(0.0060)*	*(0.0109)*	*(0.0111)*
Upper middle school degree or vocational degree	0.597 ***	0.458 ***	−0.038 ***	−0.065 ***	0.104 ***
	*(0.1029)*	*(0.0559)*	*(0.0068)*	*(0.0126)*	*(0.0128)*
University degree or higher	0.700 ***	0.485 ***	−0.043 ***	−0.066 ***	0.109 ***
	*(0.1566)*	*(0.0751)*	*(0.0087)*	*(0.0166)*	*(0.0168)*
lnper_income	0.189 ***	0.124 ***	−0.011 ***	−0.016 ***	0.027 ***
	*(0.0259)*	*(0.0147)*	*(0.0016)*	*(0.0032)*	*(0.0032)*
city	−0.058	−0.147 ***	0.003	0.029 ***	−0.033 ***
	*(0.0608)*	*(0.0315)*	*(0.0037)*	*(0.0069)*	*(0.0070)*
Constant	3.247 ***	0.967 ***			
	*(0.3378)*	*(0.1696)*			
Province—Fixed Effect	Yes		Yes		
Year—Fixed Effect	Yes		Yes		
Pseudo R^2^	0.064				
Log pseudolikelihood	−18,001.183				
Observations	22,144		22,144		

Note: (1) ***, **, and * represent the significance levels of 0.1%, 1%, and 5%, respectively. (2) In columns (i) and (ii), the standard errors are reported in parentheses. In columns (iii)—(v), the delta-method standard errors are reported in parentheses. (3) As the marital status of all individuals included in this sample is “married”, the covariates do not contain the marital variable (*married*).

**Table 4:** Estimation results of the effect of the wife’s dietary knowledge on the husband’s health based on the generalized ordinal logit model.

Variables	Baseline Model		Marginal Effect		
			*health_self* = 1 Bad	*health_self* = 2 Fair	*health_self* = 3 Good
	(i)	(ii)	(iii)	(iv)	(v)
diet_knowledge	0.095 *	0.057 *	−0.005 *	−0.007	0.012 *
	*(0.0482)*	*(0.0250)*	*(0.0027)*	*(0.0054)*	*(0.0055)*
diet_knowledge_sp	−0.088	0.025	0.005	−0.010	0.005
	*(0.0487)*	*(0.0248)*	*(0.0027)*	*(0.0054)*	*(0.0054)*
Constant	2.461 ***	1.055 ***			
	*(0.4993)*	*(0.2487)*			
Covariates	Yes		Yes		
Province—Fixed Effect	Yes		Yes		
Year—Fixed Effect	Yes		Yes		
Pseudo R^2^	0.067				
Log pseudolikelihood	−8924.568				
Observations	11,276		11,276		

Note: (1) *** and * represent the significance levels of 0.1% and 5%, respectively. (2) In columns (i) and (ii), the standard errors are reported in parentheses. In columns (iii)—(v), the delta-method standard errors are reported in parentheses. (3) As the marital status of individuals included in this sample are all “married”, the covariates do not contain the marital variable *(married*).

**Table 5:** Estimation results of the effect of the husband’s dietary knowledge on the wife’s health based on the generalized ordinal logit model.

Variables	Baseline Model		Marginal Effect		
			*health_self* = 1 Bad	*health_self* = 2 Fair	*health_self* = 3 Good
	(i)	(ii)	(iii)	(iv)	(v)
diet_knowledge	0.061	0.091 ***	−0.004	−0.016 **	0.020 ***
	*(0.0451)*	*(0.0251)*	*(0.0029)*	*(0.0056)*	*(0.0056)*
diet_knowledge_sp	0.008	0.021	−0.001	−0.004	0.005
	*(0.0447)*	*(0.0250)*	*(0.0029)*	*(0.0056)*	*(0.0056)*
Constant	3.864 ***	1.014 ***			
	*(0.4764)*	*(0.2389)*			
Covariates	Yes		Yes		
Province—Fixed Effect	Yes		Yes		
Year—Fixed Effect	Yes		Yes		
Pseudo R^2^	0.063				
Log pseudolikelihood	−9027.395				
Observations	10,868		10,868		

Note: (1) *** and ** represent the significance levels of 0.1% and 1%, respectively. (2) In columns (i) and (ii), the standard errors are reported in parentheses. In columns (iii)—(v), the delta-method standard errors are reported in parentheses. (3) As the marital status of individuals included in this sample are all “married”, the covariates do not contain the marital variable (*married*).

**Table 6:** Estimation results of the heterogeneous marginal effects of dietary knowledge on health based on the generalized ordinal logit model.

Explanatory Variable	Dependent Variable	Education		Income		Region	
		Low-Education (i)	High-Education (ii)	Low-Income (iii)	High-Income (iv)	Rural (v)	Urban (vi)
diet_knowledge	*health_self* = 1	−0.011 ***	−0.002	−0.010 ***	−0.005 *	−0.009 ***	−0.004
		*(0.0019)*	*(0.0021)*	*(0.0022)*	*(0.0018)*	*(0.0018)*	*(0.0024)*
	*health_self* = 2	−0.018 ***	−0.009	−0.013 ***	−0.018 ***	−0.023 ***	0.004
		*(0.0034)*	*(0.0051)*	*(0.0040)*	*(0.0040)*	*(0.0035)*	*(0.0048)*
	*health_self* = 3	0.029 ***	0.011 *	0.023 ***	0.022 ***	0.032 ***	−0.00004
		*(0.0034)*	*(0.0052)*	*(0.0040)*	*(0.0041)*	*(0.0035)*	*(0.0049)*
Covariates		Yes	Yes	Yes	Yes	Yes	Yes
Province—Fixed Effect		Yes	Yes	Yes	Yes	Yes	Yes
Year—Fixed Effect		Yes	Yes	Yes	Yes	Yes	Yes
Observations		19,528	9161	14,344	14,345	18,004	10,685

Note: (1) *health_self* = 1, *health_self* = 2, *health_self* = 3 represent the self-rated health status of “Bad”, “Fair “and “Good”, respectively. (2) *** and * represent the significance levels of 0.1% and 5%, respectively. (3) The coefficients in the table represent the estimated marginal effects. Delta-method standard errors are reported in parentheses.

## Data Availability

The datasets used and analyzed during the current study are available from the corresponding author on request.

## Appendix A

**Table A1:** Dietary knowledge questions and corresponding answers in the China Health and Nutrition Survey (CHNS).

Do you Strongly Agree, Somewhat Agree, Somewhat Disagree or Strongly Disagree with This Statement? 1. Strongly Disagree; 2. Disagree; 3. Neutral; 4. Agree; 5. Strongly Agree; 9. Unknown * Please Note That the Question is not Asking about Your Actual Habits	True/False
Statement	
Choosing a diet with a lot of fresh fruits and vegetables is good for one’s health.	T
Eating a lot of sugar is good for one’s health.	F
Eating a variety of foods is good for one’s health.	T
Choosing a diet high in fat is good for one’s health.	F
Choosing a diet with a lot of staple foods [rice and rice products and wheat and wheat products is not good for one’s health.	T
Consuming a lot of animal products daily (fish, poultry, eggs and lean meat) is good for one’s health.	F
Reducing the amount of fatty meat and animal fat in the diet is good for one’s health.	T
Consuming milk and dairy products is good for one’s health.	T
Consuming beans and bean products is good for one’s health.	T
Physical activities are good for one’s health.	T
Sweaty sports or other intense physical activities are not good for one’s health.	T
The heavier one’s body is, the healthier he or she is.	F

**Table A2:** Robustness check of the effect of dietary knowledge on health I (changing the calculation method of dietary knowledge).

Variables	Part A			Part B		
	Baseline Model		Marginal Effect	Baseline Model		Marginal Effect
	(i)	(ii)	(iii)	(iv)	(v)	(vi)
diet_knowledge	0.133 ***	0.129 ***	0.028 ***	0.117 ***	0.114 ***	0.025 ***
	*(0.0218)*	*(0.0131)*	*(0.0029)*	*(0.0324)*	*(0.0186)*	*(0.0041)*
diet_knowledge_sp				−0.074 *	0.007	0.002
				*(0.0337)*	*(0.0184)*	*(0.0041)*
Constant	3.017 ***	0.860 ***		3.269 ***	0.983 ***	
	*(0.2959)*	*(0.1495)*		*(0.3369)*	*(0.1690)*	
Covariates	Yes		Yes	Yes		Yes
Province—Fixed Effect	Yes		Yes	Yes		Yes
Year—Fixed Effect	Yes		Yes	Yes		Yes
Pseudo R^2^	0.071			0.064		
Log pseudolikelihood	−23,284.101			−18,038.650		
Observations	28,749		28,749	22,216		22,216

Note: (1) *** and * represent the significance levels of 0.1% and 5%, respectively. (2) In columns (i), (ii), (iv), and (v), the standard errors are reported in parentheses. In columns (iii) and (vi), the delta-method standard errors are reported in parentheses. (3) The marginal effect reported in columns (iii) and (vi) is the influence of dietary knowledge on self-rated health status as “Good”. (4) As the marital status of the Part B sample is “married”, the covariates do not contain the marital variable (*married*).

**Table A3:** Robustness check of the effect of dietary knowledge on health II (changing the sample).

Variables	Part A			Part B		
	Baseline Model		Marginal Effect	Baseline Model		Marginal Effect
	(i)	(ii)	(iii)	(iv)	(v)	(vi)
diet_knowledge	0.126 ***	0.111 ***	0.024 ***	0.077	0.074 ***	0.016 ***
	*(0.0282)*	*(0.0141)*	*(0.0030)*	*(0.0403)*	*(0.0193)*	*(0.0042)*
diet_knowledge_sp				−0.025	0.039 *	0.008 *
				*(0.0403)*	*(0.0192)*	*(0.0042)*
Constant	2.454 ***	0.630 ***		2.743 ***	0.802 ***	
	*(0.3725)*	*(0.1641)*		*(0.4190)*	*(0.1861)*	
Covariates	Yes		Yes	Yes		Yes
Province—Fixed Effect	Yes		Yes	Yes		Yes
Year—Fixed Effect	Yes		Yes	Yes		Yes
Pseudo R^2^	0.069			0.064		
Log pseudolikelihood	−19,053.292			−14,625.540		
Observations	24,809		24,809	19,023		19,023

Note: (1) *** and * represent the significance levels of 0.1% and 5%, respectively. (2) In columns (i), (ii), (iv), and (v), the standard errors are reported in parentheses. In columns (iii) and (vi), the delta-method standard errors are reported in parentheses. (3) The marginal effect reported in columns (iii) and (vi) is the influence of dietary knowledge on self-rated health status as “Good”. (4) As the marital status of the Part B sample is “married”, the covariates do not contain the marital variable (*married*).

**Table A4:** Robustness check of the effect of dietary knowledge on health Ⅲ (estimating with the reduced model without covariates *smoking* and *alcohol*).

Variables	Baseline Model		Marginal Effect		
			*health_self* = 1 Bad	*health_self* = 2 Fair	*health_self* = 3 Good
	(i)	(ii)	(iii)	(iv)	(v)
diet_knowledge	0.114 ***	0.102 ***	−0.007 ***	−0.015 ***	0.022 ***
	*(0.0233)*	*(0.0131)*	*(0.0014)*	*(0.0028)*	*(0.0029)*
gender	0.157 ***	0.210 ***	−0.010 ***	−0.037 ***	0.046 ***
	*(0.0493)*	*(0.0258)*	*(0.0031)*	*(0.0056)*	*(0.0056)*
ethnic	−0.079	0.165 ***	0.005	−0.041 ***	0.036 ***
	*(0.0875)*	*(0.0460)*	*(0.0054)*	*(0.0102)*	*(0.0101)*
age	−0.040 ***	−0.033 ***	0.002 ***	0.005 ***	−0.007 ***
	*(0.0018)*	*(0.0010)*	*(0.0001)*	*(0.0002)*	*(0.0002)*
married	0.095	0.017	−0.006	0.002	0.004
	*(0.0641)*	*(0.0374)*	*(0.0040)*	*(0.0081)*	*(0.0082)*
education					
Lower middle school degree and below	0.358 ***	0.261 ***	−0.025 ***	−0.034 ***	0.059 ***
	*(0.0686)*	*(0.0420)*	*(0.0053)*	*(0.0095)*	*(0.0096)*
Upper middle school degree or vocational degree	0.615 ***	0.420 ***	−0.040 ***	−0.055 ***	0.094 ***
	*(0.0914)*	*(0.0493)*	*(0.0060)*	*(0.0110)*	*(0.0112)*
University degree or higher	0.732 ***	0.481 ***	−0.045 ***	−0.062 ***	0.108 ***
	*(0.1399)*	*(0.0653)*	*(0.0077)*	*(0.0144)*	*(0.0145)*
lnper_income	0.175 ***	0.123 ***	−0.011 ***	−0.016 ***	0.027 ***
	*(0.0217)*	*(0.0126)*	*(0.0014)*	*(0.0027)*	*(0.0028)*
city	−0.083	−0.136 ***	0.005	0.025 ***	−0.030 ***
	*(0.0523)*	*(0.0278)*	*(0.0032)*	*(0.0061)*	*(0.0061)*
Constant	3.191 ***	0.878 ***			
	*(0.2961)*	*(0.1498)*			
Province—Fixed Effect	Yes		Yes		
Year—Fixed Effect	Yes		Yes		
Pseudo R^2^	0.069				
Log pseudolikelihood	−23,302.611				
Observations	28,689		28,689		

Note: (1) *** represents the significance level of 0.1%. (2) In columns (i) and (ii), the standard errors are reported in parentheses. In columns (iii)—(v), the delta-method standard errors are reported in parentheses.
